# Efficacy and safety of chemotherapy combined with different doses of IL-2 maintenance therapies for acute myeloid leukemia: A protocol for a Bayesian network meta-analysis

**DOI:** 10.1097/MD.0000000000026098

**Published:** 2021-06-11

**Authors:** Xuewei Yin, Yi Ding, Liming Yu, Chenchen Guo, Yanyan Cui, Xixi Zhai, Yan Wang, Shumin Ding, Mingyue Shen, Zonghong Li, Ruirong Xu

**Affiliations:** a Shandong University of Traditional Chinese Medicine; b The Second Affiliated Hospital of Shandong University of Traditional Chinese Medicine; c The Third Affiliated Hospital of Shandong First Medical University; d Neck-Shoulder and Lumbocrural Pain Hospital Affiliated to Shandong First Medical University; e Liaocheng People's Hospital; f Affiliated Hospital of Shandong University of Traditional Chinese Medicine; g Shanxi Medical University, Jinan, Shandong, China.

**Keywords:** AML, Bayesian, chemotherapy, IL-2, network meta-analysis, protocol

## Abstract

**Background:**

Acute myeloid leukemia (AML) is the most common malignant tumor of the hematopoietic system, which seriously threatens the lives of patients. Most AML patients have acute onset, severe condition, and poor prognosis. The present study aimed to comprehensively evaluate the effectiveness and safety of chemotherapy combined with different doses of interleukin-2 (IL-2) maintenance treatments in AML by Bayesian network meta-analysis (NMA).

**Methods:**

From its inception until October 2021, we will search PubMed, Cochrane Library, CNKI, Embase, and other databases to comprehensively collect randomized controlled trials (RCTs) of chemotherapy combined with different doses of IL-2 maintenance therapies for AML. Two independent researchers will complete the literature screening and data extraction according to the inclusion and exclusion criteria, and then independently conduct a bias risk assessment of all the evidence. Bayesian NMA was used to evaluate all the evidence comprehensively. Use STATA16.0 and WinBUGS1.4.3 software to process and analyze all data, and classify the quality of evidence in NMA according to grading of recommendations assessment, development, and evaluation.

**Results:**

The study will evaluate the efficacy and safety of chemotherapy combined with different doses of IL-2 maintenance therapies for AML.

**Conclusion:**

The study will provide a basis for the efficacy and safety of chemotherapy combined with different doses of IL-2 maintenance therapies for AML. We hope that this study can provide meaningful support for clinicians and patients.

**Protocol registration number:**

INPLASY202140106

**Ethical approval:**

Since the study is based on published or registered RCTs, ethical approval and patient informed consent are abandoned.

## 1 Introduction

Acute myeloid leukemia (AML) is a malignant tumor disease of the body's hematopoietic system. Although extensive research has been conducted to identify and discover prognostic markers, the mortality rate of AML is still high.^[1]^ To improve safety, low-dose chemotherapy regimens have been considered in clinical practice, but the use of low-dose drugs for chemotherapy is difficult to control the condition of AML patients at the same time, unable to achieve the expected curative effect.^[2]^

In the clinical treatment of AML, the application of adjuvant treatments can effectively reduce the adverse reactions caused by chemotherapy. Interleukin-2 (IL-2) is an immune activator, which has a significant regulatory function of immune effector cells.^[3]^ The patient was treated with high-dose IL-2 and the tumor disappeared after only a few months.^[4]^ High dose of IL-2 (12∼24 MIU/m^2^) maintenance therapy can alleviate the condition of patients with advanced AML, but its adverse reactions are also very serious.^[5]^ Pautas et al^[6]^ compared the efficacy of IL-2 with placebo in the maintenance treatment of AML through many randomized controlled trials (RCTs). However, due to the small sample size and low test efficiency of various studies, there is still a lack of large-scale, multicenter RCTs to verify its efficacy and safety. The study will use NMA to analyze and evaluate the best treatment regimen of chemotherapy combined with different doses of IL-2 maintenance therapies for AML, and provide an objective basis and medication reference for clinical diagnosis and treatment.

## 2 Methods

The study will strictly conform to the Preferred Reporting Items for Systematic Review and Meta-Analysis Protocols (PRISMA-P) specifications of the report.^[7]^

### 2.1 Study registration

The network meta-analysis (NMA) has been registered on the International Platform of Registered Systematic Review and Meta-Analysis Agreement (INPLASY), and the registration number is INPLASY202140106 (DOI number = 10.37766/inplasy2021.4.0106, URL= https://inplasy.com/inplasy-2021-4-0106/).

### 2.2 Inclusion criteria

#### 2.2.1 Type of study

The study will contain all relevant RCTs and systematic review/meta-analysis of different doses of IL-2 maintenance therapy for AML. Case reports, conference papers, overview, animal study, non-RCT, or semi-RCT trials, will not be included in the study, and the language will be confined to Chinese or English.

#### 2.2.2 Types of participants

(1) According to the clinical symptoms of patients, cytogenetics, and molecular biology, bone marrow aspiration biopsy conform to the relevant diagnostic criteria of AML,^[8]^ and confirmed the type of leukemia after flow cytometry.
(2) The diagnosis of each study group is clear, all of them are primary patients.
(3) The data included in the research are reliable and the sample size was clear.
(4) Select 1 of the duplicate documents or reports on the same population.
(5) There are no restrictions on race, nationality, gender, age, or region.
(6) The blood routine is normal and the estimated life cycle was more than 3 months.
(7) The functions of important organs are basically normal.
(8) Patients with other complex and serious diseases are not included. No previous history of coagulopathy and other tumor diseases;

#### 2.2.3 Interventions and comparisons

The intervention group was treated with conventional chemotherapy as the control group, and at the same time, various adjuvant therapies such as different doses of IL-2 were used on the basis of conventional treatment. The chemotherapy regimen: combined chemotherapy with daunorubicin, cytarabine, and homoharringtonine. In the control group, the patients were induced by general chemotherapy. In actual treatment, to ensure that the vital signs of patients are normal, the control group and intervention group need to take drugs that protect organs and provide nutrition in accordance with conventional treatment, and perform regular blood tests at the same time. The number of blood tests are ≥2 times per week and observation Patients are prescribed medications for clinical symptoms.

#### 2.2.4 Outcomes

Observation indicators: overall survival (OS) and relapse-free survival time (RFs).

Secondary outcomes: including physical signs, peripheral blood, bone marrow, electrocardiogram (ECG) and other contents, as well as factors related to myocardial enzyme spectrum, liver and kidney function, as well as the incidence of toxicity and side effects. The clinical remission effect was divided into complete remission, partial remission, and no remission.

### 2.3 Exclusion criteria

(1) The identification of AML does not meet the criteria of this article.
(2) The patients with AML who have been diagnosed and treated or experienced radiotherapy and chemotherapy, secondary AML, central nervous system, and mixed cell leukemia.
(3) A history of other malignant tumors in the past 3 years.
(4) Lack of the literature on main research indicators.
(5) Literature of qualitative research.
(6) Literature review without control study.
(7) Literature with incomplete or false clinical data.
(8) No support for follow-up treatment.
(9) Combined with heart, lung, liver, and other functional lesions.
(10) Those who have an allergy history and allergic constitution to biological products, pregnant women, and lactating women.

### 2.4 Databases and search strategy

We will carefully discuss the retrieval skills and precautions of the literature retrieval, and formulate the final retrieval strategy after multiple pre-searches. The search databases are as follows: Cochrane Library, PubMed, Cochrane Controlled Trial Center Registration, Chinese Biomedical Literature Database (SinoMed), EMBASE, CNKI, Chinese Journal Full-text Database, Wanfang Database, Chinese Science and Technology Journal Full-text Database, ScienceNet, and VIP Database. The search strategy consists of grid and keywords. The specific retrieval scheme of PubMed database is shown in Table 1.

**Table 1:** The specific retrieval scheme of PubMed database.

No.	Search item
#1	acute myeloid leukemia [MeSH Terms]
#2	acute myeloid leukemia [Title/Abstract] OR AML [Title/Abstract] OR acute, myeloid, leukemia[Title/Abstract] OR leukemia, acute, [Title/Abstract] OR leukemia, myeloid [Title/Abstract] OR leukemia [Title/Abstract]
#3	#1 OR #2
#4	chemotherapy Therapies [MeSH Terms]
#5	Therapies, chemotherapy [Title/Abstract] OR Therapy, chemotherapy [Title/Abstract] OR chemotherapy, Medicine [Title/Abstract] OR Medicine, chemotherapy [Title/Abstract] OR Demethoxydaunor Ubicin [Title/Abstract] OR Idarubicin [Title/Abstract] OR Cytosine 1-beta-D-arabinofuranoside [Title/ Abstract] OR 1-b-D-Arabinofuranosylcytosine [Title/Abstract] OR cytarabine [Title/Abstract] OR cytosine arabinoside [Title/Abstract] arabinocytidine [Title/Abstract]
#6	#4 OR #5
#7	Interleukin-2 [MeSH Terms]
#8	Interleukin-2 [Title/Abstract] OR IL-2 [Title/Abstract] OR Interleukin, 2 [Title/Abstract] OR Therapies, Interleukin-2 [Title/Abstract] OR Therapy, Interleukin-2 [Title/Abstract] OR Therapy, IL-2 [Title/Abstract] OR Therapies, IL-2 [Title/Abstract] OR Interleukin-2, Therapies [Title/Abstract] OR Interleukin-2, Therapy [Title/Abstract] OR IL-2, Therapy [Title/Abstract] OR IL-2, Therapies [Title/Abstract]
#9	#7 OR #8
#10	Randomized Controlled Trial [Publication Type] OR RCT [Publication Type] OR Controlled Clinical Trial [Publication Type] OR randomly [Title/Abstract] OR Randomized [Title/Abstract] OR random allocation [Title/Abstract]
#11	#3 AND #6 AND #9 AND #10

### 2.5 Data extraction

According to the above-mentioned search strategy, all relevant documents searched in the database were imported into EndNoteX9, and 2 researchers independently perform document screening at the same time. The controversial literature are discussed to decide whether it should be included or not. Or, if necessary, a third-party researcher can help to solve the problem and explain the reasons. Use Microsoft Excel 2019 software to record all data. The extracted literature data are as follows.

#### 2.5.1 Basic information for information release and data extraction

Title, first author, journal, publication date, country, data extraction date, title, author, journal, year, trial registration number, etc.

#### 2.5.2 Basic information of participants

Sample size, race, gender, age, source of research subjects, inclusion criteria, exclusion criteria, duration, and missing persons, etc.

#### 2.5.3 Intervention measures

Specific treatment methods, intervention details, frequency, dosage, treatment duration, etc.

#### 2.5.4 Outcome measures

Main observation indicators: OS and RFs, secondary outcomes: (physical signs, peripheral blood, bone marrow, ECG, myocardial enzymes, liver and kidney function, and the incidence of toxicity and side effects), odds ratio (OR), average deviation, confidence interval (CI), incidence of adverse events, economic cost, etc.

### 2.6 Risk of bias assessment

Two researchers will independently evaluate the methodological quality of the included RCT based on the bias risk assessment tool of Cochrane Collaboration,^[9]^ which mainly includes 7 aspects. It includes random sequence generation method, allocation concealment, blind method, result data integrity, selective result reporting bias, and other bias. Each aspect is divided into “yes,” “no” and “not clear.” If 2 researchers have different opinions, it is determined by discussing with the third researcher or contacting the original author.

### 2.7 Assessment of heterogeneity

We will apply the chi-square test to estimate the heterogeneity and use i2 statistics to assess the heterogeneity of each pair of comparisons. When i2 < 50%, the heterogeneity is small, we will use the fixed effects model, when i2 > 50%, the heterogeneity is obvious, we will use the random-effects model.

#### 2.7.1 Subgroup analysis

We will conduct a subgroup analysis of the reasons for the heterogeneity according to the source of the heterogeneity. In addition, we will use several aspects such as treatment type, disease course, age, gender, country, publication year, onset time, and duration for group analysis in different design schemes.

### 2.8 Sensitivity analysis

We will also use the exclusion method to analyze the sensitivity of all outcome indicators to disease. After changing some important factors that may affect the results, if the heterogeneity changes, then this article is the cause of the heterogeneity. On the contrary, the quality has not changed, indicating that the sensitivity is low, and the result is stable and credible.

### 2.9 Statistical analysis

#### 2.9.1 Pairwise meta-analysis

We will conduct a pairwise meta-analysis by using STATA16.0 software. Bivariate and continuous variables are represented by OR and mean difference , respectively. Analyze the 95% CI of each effect index, and evaluate the degree of heterogeneity of each pair of studies by calculating I 2.

#### 2.9.2 Network meta-analysis

We will use STATA16.0 for NMA, and use a random-effects model to merge the data and draw a network diagram. NMA uses Bayesian Markov-chain Monte Carlo method for operation, so we will apply the MCMC in WinBUGS1.4.3 to perform the Bayesian NMA of the random-effects model.^[10]^ And we will use the Brooks Gelman-Rubin statistical method to assess convergence. If the potential proportional reduction factor tends to 1, it means that the convergence of the model is more reliable.^[11,12]^ We will use the surface under the cumulative ranking curve value to rank interventions.^[13]^ The surface under the cumulative ranking cure value ranges from 0 to 1. When the value is close to 1, the convergence is better and the conclusion is more credible, and the more likely the intervention becomes the best intervention. When there is a closed loop in NMA, we need to evaluate its consistency.^[14]^ Therefore, we will use the node segmentation method to calculate the difference between the direct comparison evidence and the indirect comparison evidence, and then we use the *P* value to determine whether there is any inconsistency.^[15,16]^

#### 2.9.3 Assessment of publication bias

For articles with more than 10 studies, a funnel chart is used to analyze the publication bias for potential publication bias. Publication bias can be evaluated by observing the symmetry of the funnel plot. If the funnel is symmetric, there is no obvious publication bias. If it is asymmetric, there may be a release bias, and then we will analyze the reason.

#### 2.9.4 Assessment of the quality of evidence

We will evaluate the quality of evidence and the strength of recommendations through the grading of recommendations assessment, development, and evaluation (GRADE) method. The evaluation of GRADE in the NMA mainly includes 5 aspects: research limitations, inconsistencies, indirectness, publication bias, and inaccuracy.^[17]^ GRADE divides the quality of evidence into 4 levels: high, medium, low, and very low. The recommended strength is divided into 2 levels: strong and weak.

## 3 Discussion

In recent years, bone marrow suppression is obvious after receiving high-dose chemotherapy in clinical AML patients, the immune function decreased, and the prognosis of patients was relatively higher poor.^[18–21]^ IL-2 can treat AML by activating and expanding functional NK cells and T cells in the body.^[22]^ IL-2 may be more effective for young AML patients, because young AML patients are more sensitive to chemotherapy, and young people are more willing to receive more intense induction chemotherapy and adjuvant therapy.^[23]^ Donor lymphocyte infusion combined with IL-2 and other cytokines have good prospects for the treatment of malignant tumors.^[24]^

NMA is based on an indirect comparative meta-analysis or multiple interventions, which has obvious advantages. In this study, we will use Bayesian NMA to compare the effectiveness and safety of different chemotherapy combined with different doses of IL-2 maintenance therapies. The interventions were ranked through SURCA, and GRADE was used to assess the quality of the evidence. In this study, although we conducted NMA using the Bayesian model, our study still has some limitations. For example, there may be an uneven quality of literary research in our research materials, which reduces the reliability of the research. Therefore, we hope to include more high-quality, large-scale, multi-center RCTs to continuously improve the level of evidence-based medicine. We hope that this study can help clinicians and patients to choose the best scheme to improve the efficacy and safety of chemotherapy combined with different doses of IL-2 maintenance therapies for AML, and provide a better decision-making basis for clinical practice.

## Author contributions

**Conceptualization:** Xuewei Yin, Ruirong Xu.

**Data curation:** Yi Ding, Liming Yu.

**Investigation:** Xuewei Yin, Yi Ding, Chenchen Guo.

**Methodology:** Yanyan Cui, Xixi Zhai, Yan Wang.

**Software:** Mingyue Shen, Zonghong Li.

**Validation:** Yi Ding, Shumin Ding.

**Writing – original draft:** Xuewei Yin, Liming Yu.

**Writing – review & editing:** Xuewei Yin, Yi Ding, Ruirong Xu.

## References

[1] Döhner H Estey E Grimwade D. Diagnosis and management of AML in adults: 2017 ELN recommendations from an international expert panel. Blood 2017;129:424. 27895058 10.1182/blood-2016-08-733196 PMC5291965

[2] Qu Q Liu L Zhang Y Li X Wu D. Increasing aclarubicin dosage of the conventional CAG (low-dose cytarabine and aclarubicin in combination with granulocyte colony-stimulating factor) regimen is more efficacious as a salvage therapy than CAG for relapsed/refractory acute myeloid leukemia. Leuk Res 2015;39:1353–9. 26432074 10.1016/j.leukres.2015.09.014

[3] Wang Z-H Dai G Zhou R-L Kuang Z-M. Effects of recombinant human interleukin-2 activated natural killer cells on cardiac function in rats with myocardial infarction. Zhonghua Xin Xue Guan Bing Za Zhi 2013;41:778–84. 24331808

[4] Rosenberg SA Lotze MT Muul LM. Observations on the systemic administration of autologous lymphokine-activated killer cells and recombinant interleukin-2 to patients with metastatic cancer. N Engl J Med 1985;313:1485–92. 3903508 10.1056/NEJM198512053132327

[5] Kolitz JE George SL Benson DM. Recombinant interleukin-2 in patients aged younger than 60 years with acute myeloid leukemia in first complete remission: results from Cancer and Leukemia Group B 19808. Cancer 2013;120:1010–7. 24382782 10.1002/cncr.28516 PMC4139069

[6] Pautas C Merabet F Thomas X. Randomized study of intensified anthracycline doses for induction and recombinant interleukin-2 for maintenance in patients with acute myeloid leukemia age 50 to 70 years: results of the ALFA-9801study. J Clin Oncol 2010;28:808–14. 20048183 10.1200/JCO.2009.23.2652

[7] Shamseer L Moher D Clarke M. Preferred reporting items for systematic review and meta-analysis protocols (PRISMA-P) 2015: elaboration and explanation. BMJ 2015;350:g7647. 25555855 10.1136/bmj.g7647

[8] Zhang Zhinan Shen Ti. Diagnostic and therapeutic criteria of hematological diseases. 2007;Beijing: Science Press, 131-132.

[9] Cumpston M Li T Page MJ. Updated guidance for trusted systematic reviews: a new edition of the Cochrane Handbook for Systematic Reviews of Interventions. Cochrane Database Syst Rev 2019;10:01–2. 10.1002/14651858.ED000142 PMC10284251 31643080

[10] Lunn DJ Thomas A Best N Spiegelhalter D. WinBUGS: a Bayesian modelling framework: concepts, structure, and extensibility. Stat Comput 2000;10:325–37.

[11] Gelman BA. General methods for monitoring convergence of iterative simulations. J Comput Graph Stat 1998;7:434–55.

[12] Burger DA Schall R. A Bayesian nonlinear mixed-effects regression model for the characterization of early bactericidal activity of tuberculosis drugs. J Biopharm Stat 2015;25:1247–71. 25322214 10.1080/10543406.2014.971170 PMC4673548

[13] Salanti G Ades AE Ioannidis JPA. Graphical methods and numerical summaries for presenting results from multiple-treatment meta-analysis: an overview and tutorial. J Clin Epidemiol 2011;64:163–71. 20688472 10.1016/j.jclinepi.2010.03.016

[14] Dias S Welton NJ Caldwell DM Ades AE. Checking consistency in mixed treatment comparison meta-analysis. Stat Med 2010;29:932–44. 20213715 10.1002/sim.3767

[15] Albert I Makowski D. Ranking crop species using mixed treatment comparisons. Res Synth Methods 2019;10:343–59. 30353974 10.1002/jrsm.1328

[16] Sauter R Held L. Network meta-analysis with integrated nested Laplace approximations. Biom J 2015;57:1038–50. 26360927 10.1002/bimj.201400163

[17] Puhan MA Schunemann HJ Murad MH. A GRADE Working Group approach for rating the quality of treatment effect estimates from network meta-analysis. BMJ 2014;349:g5630. 25252733 10.1136/bmj.g5630

[18] Ammann RA Laws HJ Schrey D. Bloodstream infection in paediatric cancer centres-leukaemia and relapsed malignancies are independent risk factors. Eur J Pediatr 2015;174:675–86. 25804192 10.1007/s00431-015-2525-5

[19] Kato H Fujita H Akiyama N. Infectious complications in adults undergoing intensive chemotherapy for acute myeloid leukemia in 2001–2005 using the Japan Adult Leukemia Study Group AML201 protocols. Support Care Cancer 2018;26:4187–98. 29860713 10.1007/s00520-018-4292-0

[20] Goswami M Prince G Biancotto A. Impaired B cell immunity in acute myeloid leukemia patients after chemotherapy. J Transl Med 2017;15:155. 28693586 10.1186/s12967-017-1252-2 PMC5504716

[21] Halpern AB Culakova E Walter RB Lyman GH. Association of risk factors, mortality, and care costs of adults with acute myeloid leukemia with admission to the intensive care unit. JAMA Oncol 2017;3:374–81. 27832254 10.1001/jamaoncol.2016.4858 PMC5344736

[22] Luo S Cai F Jiang L. Clinical study of Mito-FLAG regimen in treatment of relapsed acute myeloid leukemia. Exp Ther Med 2013;5:982–6. 23407597 10.3892/etm.2013.917 PMC3570250

[23] Baer MR George SL Caligiuri MA. Low-dose interleukin-2immunotherapy does not improve outcome of patients age 60 years and older with acute myeloid leukemia in first complete remission: Cancer and Leukemia Group B Study 9720. J Clin Oncol 2008;26:4934–9. 18591543 10.1200/JCO.2008.17.0472 PMC2652081

[24] Gesundheit B Shapira MY Resnick IB. Successful cell-mediated cytokine-activated immunotherapy for relapsed acute myeloid leukemia after hematopoietic stem cell transplantation. Am J Hematol 2009;84:188–90. 19105234 10.1002/ajh.21346

